# Evaluation of the Revised Ceftaroline Disk Diffusion Breakpoints When Testing a Challenge Collection of Methicillin-Resistant Staphylococcus aureus Isolates

**DOI:** 10.1128/JCM.00777-18

**Published:** 2018-11-27

**Authors:** Helio S. Sader, Paul R. Rhomberg, Timothy B. Doyle, Robert K. Flamm, Rodrigo E. Mendes

**Affiliations:** a JMI Laboratories, North Liberty, Iowa, USA; Medical College of Wisconsin

**Keywords:** MRSA, ceftaroline, disk diffusion

## Abstract

We assessed ceftaroline disk diffusion breakpoints for Staphylococcus aureus when applying revised Clinical and Laboratory Standards Institute (CLSI) ceftaroline MIC breakpoints. Disk-MIC correlation was evaluated by testing a challenge collection (*n* = 158) of methicillin-resistant S. aureus (MRSA) isolates composed of 106 randomly selected isolates plus 52 isolates with decreased susceptibility to ceftaroline (MIC, 1 to 16 μg/ml). Disk diffusion was performed with 30-μg disks and Mueller-Hinton agar from 2 manufacturers each. Revised CLSI susceptible (S)/susceptible dose-dependent (SDD)/resistant (R) MIC breakpoints of ≤1/2 to 4/≥8 μg/ml were applied. The disk breakpoints that provided the lowest error rates were CLSI S/R breakpoints of ≥25 mm/≤19 mm, with no very major (VM) or major (Ma) errors and with minor (Mi) error rates of 0.0% for ≥2 doubling dilutions above the I or SDD (≥I + 2), 22.1% for I or SDD plus or minus 1 doubling dilution (I ± 1), and 2.3% for ≤2 doubling dilutions below the I or SDD ≤I − 2 (overall Mi error rate, 16.5%). No mutation in the penicillin-binding protein 2a (PBP2a) was observed in 5 of 15 isolates with a ceftaroline MIC of 2 μg/ml; 3 of 11 isolates with a ceftaroline MIC of 1 μg/ml exhibited mutations in the penicillin-binding domain (PBD; 1 isolate) or in the non-PBD (2 isolates). All isolates except 1, with a ceftaroline MIC of ≥4 μg/ml, showed ≥1 mutation in the PBD and/or non-PBD. In summary, results from the disk diffusion method showed a good correlation with those from the reference broth microdilution method. Our results also showed that the ceftaroline MIC distribution of isolates with no mutations in the PBP2a goes up to 4 μg/ml, and reference broth microdilution and disk diffusion methods do not properly separate wild-type from non-wild-type isolates.

## Abstract (precis)

We assessed ceftaroline disk diffusion breakpoints for Staphylococcus aureus when applying revised Clinical and Laboratory Standards Institute (CLSI) ceftaroline MIC breakpoints. Disk-MIC correlation was evaluated by testing a challenge collection (*n* = 158) of methicillin-resistant S. aureus (MRSA) isolates composed of 106 randomly selected isolates plus 52 isolates with decreased susceptibility to ceftaroline (MIC, 1 to 16 μg/ml).

## INTRODUCTION

Categorical breakpoints of antimicrobial susceptibility tests are initially defined for reference broth microdilution or agar dilution methods by correlating microbiological and clinical outcomes with MIC data for the infecting organisms (1). According to the Clinical and Laboratory Standards Institute (CLSI) guidelines (2), disk diffusion breakpoints are established after MIC breakpoints have been determined by plotting a scattergram of the zone of inhibition diameters versus MIC values for isolates tested by both methods. Because of the inherent variation of these susceptibility testing methods, the correlation between MIC and zone diameter is not completely linear. Thus, the zone of inhibition diameter interpretative criteria that provide the lowest intermethod error rates (or discrepancy rates) are selected using a statistical method, the denominated error rate-bounded method (1, 3, 4). It is important to note that the discrepancy rates, i.e., very major (VM), major (Ma), and minor (Mi) error rates, are directly proportional to the percentage of isolates with MIC values in the range of plus or minus 1 doubling dilution of the breakpoints (2). Therefore, the performance of the disk diffusion test will vary according to the susceptibility of the organism collection to the antimicrobial agent being tested.

Methicillin resistance in Staphylococcus aureus is mediated by the horizontal acquisition of the staphylococcal cassette chromosome (SCC) that carries the *mecA* allele, which encodes an alternative penicillin-binding protein 2a (PBP2a). β-Lactam compounds act by binding to “native” PBPs and inhibiting the transpeptidation reaction required during peptidoglycan synthesis. However, most β-lactam molecules have a poor binding affinity for PBP2a, which, once acquired, functionally complement the transpeptidation reaction and allows cell wall biogenesis to proceed in the presence of these β-lactams (5, 6).

The SCC *mec* element (SCC*mec*) plays an important role in the antimicrobial resistance characteristics, molecular epidemiology, and evolution of methicillin-resistant S. aureus (MRSA). The most common SCC*mec* types reported worldwide are types I to V. The SCC*mec* type influences the activity (MIC value) of the β-lactams and the resistance patterns to antimicrobial agents of other classes (7–9).

Ceftaroline fosamil, the prodrug of the active metabolite ceftaroline, is a broad-spectrum cephalosporin approved in 2010 by the U.S. Food and Drug Administration (FDA) and in 2012 by the European Medicines Agency (EMA) for the treatment of community-acquired bacterial pneumonia (CABP) and acute bacterial skin and skin structure infections (ABSSSIs). Ceftaroline has high affinity for PBP2a while maintaining high affinity for other essential S. aureus PBPs (6), and because of its high affinity to PBP2a, ceftaroline is active against MRSA. However, as for other β-lactam compounds, ceftaroline potency (MIC value) against MRSA varies according to the SCC*mec* type. Among MRSA isolates with SCC*mec* types I to IV, the lowest ceftaroline MIC values are observed with isolates carrying SCC*mec* type IV, followed by types II, III, and I (8).

Since a relatively small number of pandemic MRSA clones have caused a majority of MRSA infections worldwide and a few specific clones tend to disseminate and predominate in a geographic region (10–12), the *in vitro* activity of ceftaroline may vary by geographic region based on the SCC*mec* types of clones that predominate in a particular region. Data from the SENTRY Antimicrobial Surveillance Program for isolates consecutively collected worldwide in 2016 and 2017 show ceftaroline susceptibility (S) rates against MRSA of 95.1% in the United States, 88.7% in Europe, 77.8% in the Asia-Pacific region, and 74.7% in Latin America (Table 1). Data from the SENTRY program also show important variations in ceftaroline susceptibility rates among countries from the same region (data on file; JMI Laboratories).

**TABLE 1:** Summary of ceftaroline activity tested against MRSA stratified by geographic region in the SENTRY program, 2016 to 2017

Isolate region or group (no. tested)	No. (cumulative %) of isolates inhibited at ceftaroline MIC (μg/ml) of:							% S	% SDD	% R
	0.25	0.5	1	2	4	8	16			
North America (5,454)	177 (3.2)	2,967 (57.6)	2,042 (95.1)	265 (99.9)	3 (100.0)			95.1	4.9	0.0
Europe (1,497)	44 (2.9)	622 (44.5)	662 (88.7)	169 (100.0)				88.7	11.3	0.0
Latin America (495)	3 (0.6)	173 (35.6)	194 (74.7)	116 (98.2)	9 (100.0)			74.7	25.3	0.0
Asia-Pacific (666)	8 (1.2)	305 (47.0)	205 (77.8)	105 (93.5)	37 (99.1)	1 (99.2)	5 (100.0)	77.8	21.3	0.9
All isolates (8,112)	232 (2.9)	4,067 (53.0)	3,103 (91.2)	655 (99.3)	49 (99.9)	1 (99.9)	5 (100.0)	91.2	8.7	0.1

Although clinical efficacy data in patients with MRSA displaying a ceftaroline MIC of ≥2 μg/ml are very limited, results from more recent pharmacokinetic/pharmacodynamic (PK/PD) studies have indicated that ceftaroline MIC breakpoints should be reevaluated (13–15). Furthermore, results from a phase 3 randomized controlled noninferiority trial have shown that ceftaroline fosamil at 600 mg administered every 8 h with 2-h infusions was effective and well tolerated in the treatment of patients with complicated skin and skin structure infections and can provide adequate exposure against S. aureus, with ceftaroline MIC values of ≤4 μg/ml (13, 15, 16). Thus, the CLSI has recently reevaluated ceftaroline breakpoints based on microbiology, PK/PD, and clinical data generated after the current breakpoints were established and has changed susceptible (S)/intermediate (I)/resistant (R) MIC breakpoints from ≤1/2/≥4 μg/ml to susceptible/susceptible dose-dependent (SDD)/resistant MIC breakpoints of ≤1/2 to 4/≥8 μg/ml (17). The main objective of this investigation was to assess ceftaroline disk diffusion breakpoints for S. aureus when applying the revised CLSI ceftaroline MIC breakpoints. Secondary objectives included (i) assessing the influence of the disk and Mueller-Hinton agar reagents on the performance of the disk diffusion test and (ii) characterizing a subset of MRSA isolates and evaluating the ability of susceptibility testing methods to segregate wild-type (defined here as MRSA isolates with absence of mutations in PBP2a) from non-wild-type populations.

## MATERIALS AND METHODS

### Organism collection.

A total of 158 MRSA isolates were evaluated in this investigation. The organism collection comprised a subset of 106 MRSA isolates that were randomly selected from a collection of 1,596 MRSA clinical isolates recovered from non-U.S. countries during the SENTRY program for 2016 and a subset of 52 clinical MRSA isolates with elevated ceftaroline MIC values (1 to 16 μg/ml) that were included to provide a better evaluation of the categorical agreement between results from disk diffusion and broth microdilution methods. Ceftaroline susceptibility and resistance rates according to the revised CLSI breakpoint criteria (17) were 62.0% and 3.8%, respectively, with 34.2% of isolates categorized as susceptible dose dependent.

### Antimicrobial susceptibility testing.

Isolates were tested for susceptibility to ceftaroline by reference broth microdilution and disk diffusion methods, as described by the CLSI (18, 19), and susceptibility interpretations were based on the 2019 CLSI document M100 (17). The MIC panels were manufactured at JMI Laboratories (North Liberty, IA, USA), and the organisms were tested in cation-adjusted Mueller-Hinton broth (Becton, Dickinson and Company, Franklin Lakes, NJ, USA). Ceftaroline powder was obtained from Allergan, Inc. (Patheon API Services, Florence, SC, USA).

Each isolate was tested by the disk diffusion method using ceftaroline 30-μg disks from 2 manufacturers (disks A and B) and cation-adjusted Mueller-Hinton agar (MHA) from 2 manufacturers (MHA 1 and 2). Thus, there were 4 zone diameter values for each MIC value. Ceftaroline 30-μg disks were obtained from Hardy/MAST (disk A) and Becton Dickinson-BBL (disk B), and MHA was obtained from Remel (MHA 1) and Becton Dickinson-BBL (MHA 2). All VM, Ma, and selected minor errors were repeated. S. aureus ATCC 29213 and ATCC 25923 were tested in each experiment, and all quality control (QC) values were within the expected range.

### Data analysis.

Ceftaroline breakpoints of ≤1/2 to 4/≥8 μg/ml (S/SDD/R) for MIC and ≥25/20 to 24/≤19 mm (S/SDD/R) for disk diffusion were applied as established by the 2019 M100 CLSI document (17). Discrepancy rates between MIC values and zone diameter test results were calculated according to the CLSI M23 document (2). Discrepancies involving false-susceptible disk results were defined as VM errors, whereas false-resistant disk diffusion results were defined as Ma errors. Discrepancies involving the intermediate (I) category were defined as Mi errors. Optimal disk breakpoints were determined by the error rate-bounded method according to CLSI M23 document (2) using a software developed by JMI Laboratories based on the dBETS software (3). In brief, the proposed zone diameter breakpoints were adjusted until discrepancy rates, i.e., VM, Ma, and Mi errors, were held to a minimum (2). Error rates were considered acceptable at <10% for VM and Ma and <40% for Mi for the population of MICs in the I or SDD plus or minus 1 doubling dilution (I + 1 to I − 1, or I ± 1), at <2% for VM and <5% for Mi for the population of MICs ≥2 doubling dilutions above the I or SDD (≥I + 2), and at <2% for Ma and <5% for Mi for the population of MICs ≥2 doubling dilutions below the I or SDD (≤I − 2) (2). Differences in mean disk diffusion diameters between disk and broth types were tested using analysis of variance (ANOVA) and Tukey's honestly significant difference (HSD) test.

### Molecular characterization of selected isolates.

A total of 51 isolates were submitted for whole-genome sequencing, including all 25 isolates with ceftaroline MIC results of ≥4 μg/ml. In addition, 15 of 35 isolates with ceftaroline MIC results of 2 μg/ml and 11 of 54 isolates with a ceftaroline MIC of 1 μg/ml were randomly selected for molecular characterization. Genomes were sequenced on a MiSeq sequencer (JMI Laboratories). Genomic DNA of isolates was extracted using the Thermo Scientific KingFisher Flex magnetic particle processor (Cleveland, OH, USA) and used as input material for library construction. DNA libraries were prepared using the Nextera XT library construction protocol (Illumina, San Diego, CA, USA) according to the manufacturer's instructions. Sequences were assembled using SPAdes 9.3.0, and the assembled genomes were subjected to a proprietary pipeline to determine the SCC*mec* type and multilocus sequence type (ST). In addition, the respective *mecA*, *pbp4*, and *lytD* sequences, where alterations were previously associated with decreased susceptibility to ceftaroline, were extracted and screened for predicted amino acid alterations compared with control isolates that had the same SCC*mec* type and lineage background (i.e., ST). MRSA isolates with no mutations in the PBP2a were defined as wild-type MRSA for the purposes of this investigation (5, 6).

## RESULTS

**FIG 1:**
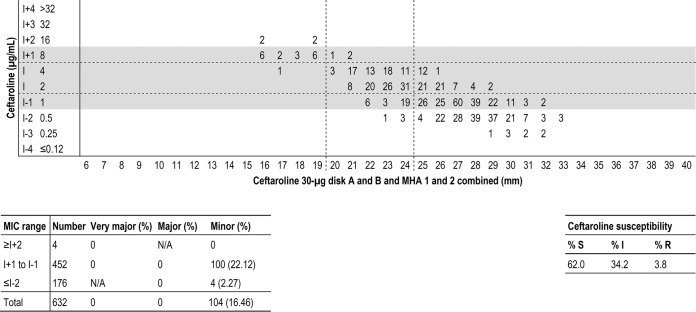
Scattergram comparing the results of ceftaroline broth microdilution MIC values (in micrograms per milliliter) and disk diffusion zone diameters (in millimeters) for a 30-μg disk when testing all MRSA isolates (632 results for 158 isolates). Each isolate was tested with ceftaroline disks from 2 manufacturers (disk A and disk B) and in Mueller-Hinton agar from 2 manufacturers (MHA 1 and MHA 2). Horizontal and vertical broken lines indicate ceftaroline breakpoints (CLSI). The table at the bottom displays the number of isolates tested and very major, major, and minor error rates for each category.

The disk breakpoints that provided the lowest error rates were ≥25 mm/≤19 mm for S/R, with no VM or Ma errors and with Mi error rates of 0.0% for ≥I + 2, 22.1% for I ± 1, and 2.3% for ≤I − 2 (overall Mi error rate, 16.5%; Fig. 1). Of note, the CLSI-acceptable minor error rates are <5% for ≥I + 2 and ≤I − 2, and <40% for I ± 1 (2). Error rates would increase slightly if either the S or the R disk breakpoint would move 1 mm upward or downward (Table 2).

**TABLE 2:** Error rates for possible 30-μg disk breakpoints when applying current CLSI MIC breakpoints^*a*^

Disk breakpoints (S/R) by zone diam (mm)^*b*^	No. of results^*c*^	Error rate (%)^*d*^		
		Very major	Major	Minor
≥24/≤18				
≥I + 2	4	0	NA	2 (50.0)
I + 1 to I − 1	452	0	0	129 (28.54)
≤I − 2	176	NA	0	1 (0.57)
Total	632	0	0	132 (20.89)
≥25/≤18				
≥I + 2	4	0	NA	2 (50.0)
I + 1 to I − 1	452	0	0	106 (23.45)
≤I − 2	176	NA	0	4 (2.27)
Total	632	0	0	112 (17.72)
≥26/≤18				
≥I + 2	4	0	NA	2 (50.0)
I + 1 to I − 1	452	0	0	99 (21.9)
≤I − 2	176	NA	0	8 (4.55)
Total	632	0	0	109 (17.25)
**≥25/≤19^*b*^**				
**≥I + 2**	**4**	**0**	**NA**	**0**
**I + 1 to I − 1**	**452**	**0**	**0**	**100 (22.12)**
**≤I − 2**	**176**	**NA**	**0**	**4 (2.27)**
**Total**	**632**	**0**	**0**	**104 (16.46)**
≥26/≤19				
≥I + 2	4	0	NA	0
I + 1 to I − 1	452	0	0	93 (20.58)
≤I − 2	176	NA	0	8 (4.55)
Total	632	0	0	101 (15.98)
≥26/≤20				
≥I + 2	4	0	NA	0
I + 1 to I − 1	452	0	0	95 (21.02)
≤I − 2	176	NA	0	8 (4.55)
Total	632	0	0	103 (16.3)

a Breakpoints of ≤1 μg/ml for susceptible, 2 to 4 μg/ml for susceptible dose dependent, and ≥8 μg/ml for resistant.

b S, susceptible; R, resistant, I, intermediate. Values in bold indicate breakpoints and error rates for current CLSI disk breakpoints (CLSI M100 [17]).

c The total of 632 refers to 4 disk results for each of the 158 isolates (4 × 158).

d NA, not applicable.

When the results were stratified according to disk and MHA manufacturers, the mean, geometric mean, and median values for zone diameters varied approximately 2 mm between disk A and disk B, independent of the MHA used, and this difference was statistically significant (*P* < 0.001). No significant difference was observed between the zone of inhibition diameter results from the 2 MHA manufacturers when using the same disk manufacturer (data not shown). Of note, all inhibition zones for QC strain S. aureus ATCC 25923 were within CLSI-acceptable ranges (26 to 35 mm), with mean values of 28.3 mm (disk A and MHA 1) to 31.7 mm (disk B and MHA 2) and an overall mean value of 29.8 mm (data not shown). Furthermore, when error rates were analyzed for each disk separately, both disks displayed acceptable error rates, with no major or very major errors, and they had minor error rates of 0.0%, 14.2%, and 4.5% for disk A and 0.0%, 29.6%, and 0.0% for disk B at ≥I + 2, I ± 1, and ≤I − 2, respectively (data not shown).

The majority of isolates selected for molecular characterization belonged to clonal complex 5 (CC5) (43/51 [84.3%]) and were associated with 8 STs (Table 3). The remaining isolates belonged to CC4 and 4 STs. Among CC5 isolates, the majority (39/43 [90.7%]) showed ceftaroline MIC values of ≥2 μg/ml (nonsusceptible), while most of those belonging to other CCs (7/8 [87.5%]) displayed ceftaroline MIC results at ≤1 μg/ml (Table 3). In addition, most isolates (26/40 [65.0%]) exhibiting ceftaroline MIC values of ≥2 μg/ml were recovered from countries in the Asia-Pacific region (Japan, South Korea, Taiwan, and Thailand).

**TABLE 3:** Distribution of MRSA lineages and country of origin according to the ceftaroline MIC value

Country of origin by clonal complex^*a*^	No. of isolates at each ceftaroline MIC (μg/ml)				
	1	2	4	8	16
CC5					
Argentina		1			
Chile		2			
Hungary	1				
Italy		2			
Japan	1	1	4		
South Korea		3	10	4	1
Mexico		1			
Peru		2	2		
Slovenia	1				
Spain	1				
Taiwan		1			
Thailand			1	1	
United States		1	2		
CC8					
Russia	2				
CC22					
Australia	1				
Ireland	1				
Italy		1			
New Zealand	1				
CC45					
Belgium	1				
CC239					
Australia	1				
Total	11	15	19	5	1

a Clonal complex 5 (CC5) represented by ST5 (29 isolates), ST105 (*n* = 2), ST125 (*n* = 1), ST228 (*n* = 3), ST518 (*n* = 1), ST764 (*n* = 5), ST1110 (*n* = 1), and ST2883 (*n* = 1); CC8, ST8; CC22, ST22; CC45, ST45; and CC239, ST239.

The most common SCC*mec* type observed among the 51 characterized isolates was type II (*n* = 34 [66.7%]), followed by types I (*n* = 9 [17.6%]), IV (*n* = 7 [13.7%]), and III (*n* = 1 [2.0%]). Ceftaroline-nonsusceptible isolates (MIC, ≥2 μg/ml) were mainly SCC*mec* types II (30 of 40 [75.0%]) and I (9 of 40 [22.5%]), whereas ceftaroline-susceptible isolates were mainly SCC*mec* types IV (6 of 11 tested [54.5%]) and II (4 of 11 tested [36.4%]; data not shown).

Table 4 shows alterations detected in the PBP2a of selected isolates. Among those isolates having a wild-type sequence for PBP2a, the ceftaroline MIC results varied between 1 and 4 μg/ml, with a modal MIC of 1 μg/ml. The ceftaroline MIC range was also 1 to 4 μg/ml when testing MRSA isolates demonstrating alterations within the allosteric site (non-penicillin-binding domain [nPBD] residues 27 to 326), with a modal MIC value of 2 μg/ml. Those MRSA isolates with alterations at the allosteric and transpeptidase (PBD) sites showed a ceftaroline modal MIC value of 4 μg/ml, as did those isolates with mutations at the transpeptidase site only. R130C alterations in LytD were observed in all MRSA isolates from South Korea (ST5-MRSA-II), and these results likely reflect a polymorphism associated with that clone. Other isolates showed wild-type sequences for LytD. Most isolates (33/51 [64.7%]) showed an upstream sequence of *pbp4* consistent with reference control strains. A total of 12 isolates had deletions within this upstream region, which was previously associated with decreased susceptibility to ceftaroline and other β-lactam agents (20). Another 6 isolates had a minor number of nucleotide alterations compared to the control strains (Table 4). In addition, isolates exhibiting ceftaroline MIC values of 4 to 16 μg/ml did not tend to have PBP2a mutations with alterations in the upstream region of *pbp4* (3/25 [12.0%]), while those MRSA isolates with MIC results of 1 to 2 μg/ml had greater chances (15/26 [57.7%]) of having alterations in both sequences (Table 4).

**TABLE 4:** Correlation of ceftaroline MIC result with alterations detected in PBP2a and the upstream region of *pbp4* among isolates selected for further molecular characterization

PBP2a wild type or mutation (CC)^*a*^	No. of isolates at each ceftaroline MIC (μg/ml)^Alteration observed at *pbp4**b*^				
	1	2	4	8	16
WT (CC5, CC8, CC22, CC45, CC239)	8^4Del,4mutations^	5^3WT,2Del^	1^WT^		
N146K (CC5, CC239)	1^WT^	1^Del^			
K565N (CC5)^*c*^	1^G105T^				
E246G (CC22)		1^Mutations^			
E150K (CC5)	1^Del^	5^1Del,4WT^	2^WT^		
N146K, L357I (CC5)		1^WT^			
N146K, L357I, I563T (CC5)		2^WT^	2^WT^		
A228V, L357I, I563T (CC5)			6^WT^		
N146K, L357I, M411I, I563T (CC5)			1^WT^		
E447K (CC5)			5^2Del,3WT^		
N104K, V470I (CC5)			1^WT^		
K146N, E239K, E447K (CC5)			1^Del^	1^WT^	
N104K, V117I, E447K, I563T, S649A (CC5)				4^WT^	1^WT^
Location of alteration in PBP2a					
Allosteric site	2	7	2		
Allosteric and transpeptidase sites		3	11	5	1
Transpeptidase site	1^*c*^		6		
Total	11	15	19	5	1

a PBP2a, penicillin-binding protein 2a; WT, wild type; allosteric site represented by amino acid residues 27 to 326; transpeptidase domain represented by residues 327 to 668. Ceftaroline breakpoints of ≤1 (susceptible)/2 to 4 μg/ml (shaded cells; susceptible dose dependent)/≥8 μg/ml (resistant) for MIC were applied as established by the CLSI (17).

b Del, deletion in the region upstream of *pbp4*; a single G105T alteration in the position 105 upstream of *pbp4*. Mutations are represented by several nucleotide alterations within 400 bp upstream of *pbp4*.

c K565N alteration at PBP2a.

When ceftaroline was tested by disk diffusion against strains showing a wild-type sequence for PBP2a, 67.9% of results were categorized as S and 32.1% as SDD (Table 5). A total of 22.7% of disk diffusion results were categorized as S and 77.3% as SDD when tested against those isolates having PBP2a alterations in the allosteric site, while 81.2% of disk diffusion results for isolates carrying PBP2a alterations at both the allosteric and transpeptidase sites and 62.5% of disk diffusion results for isolates carrying alterations at the transpeptidase site were categorized as ceftaroline nonsusceptible. Furthermore, all ceftaroline-resistant isolates (MIC, ≥8 μg/ml) exhibited alterations at both the allosteric and transpeptidase sites (Table 5).

**TABLE 5:** Correlation of ceftaroline MIC, disk zone results, and PBP2a alterations detected among isolates selected for further molecular characterization

Ceftaroline MIC (μg/ml) by PBP2a status^*b*^	No. of isolates by disk zone diam (mm)^*a*^																	Susceptibility rates (%)		
	16	17	18	19	20	21	22	23	24	25	26	27	28	29	30	31	32	S	SDD	R
WT																				
1							3	1	4		1	3	6	3	8	1	2	67.9	32.1	0.0
2							2	3	1	4	5	3	2							
4						1	1	1	1											
Allosteric site																				
1								1	3	2	2							22.7	77.3	0.0
2						5	3	5	9	2	2		2							
4						4	1	3												
Allosteric and transpeptidase sites																				
2							2	2		3	3			2				18.8	53.8	27.5
4		1			1	9	7	11	8	6	1									
8	6	2	3	6	1	2														
16	2			2																
Transpeptidase sites																				
1							1				1		2					37.5	62.5	0.0
4					2	3	4	3	2	6										

a Four zone diameter results are shown for each MIC value (2 manufacturers for ceftaroline disks and 2 manufacturers for Mueller-Hinton agar).

b WT, wild type. Allosteric site represented by amino acid residues 27 to 326; transpeptidase domain represented by residues 327 to 668. Ceftaroline breakpoints of ≥25 mm (susceptible)/24 to 20 mm (shaded cells; susceptible dose dependent)/≤19 mm (resistant) for disk diffusion method were applied as established by the CLSI (17).

## DISCUSSION

Accurate antimicrobial susceptibility testing is essential for the proper treatment of bacterial infections. Although disk diffusion testing is rarely used for routine susceptibility testing in the United States and western European countries, it is still commonly used in many geographic regions, especially in developing countries. Furthermore, disk diffusion testing represents an important tool for testing recently approved antimicrobial agents, since the inclusion of novel agents on commercial antimicrobial susceptibility systems may take several years after their approval for clinical use (21).

We evaluated the correlation between MIC and disk diffusion zone diameters using a collection of MRSA isolates heavily biased toward ceftaroline nonsusceptibility, which had 71.5% of isolates (113/158) with ceftaroline MIC values at the breakpoint dilutions (1 to 8 μg/ml). The results of this investigation showed that error rates were acceptable and generally low even when a very challenging organism collection was used. It is important to note that the zone diameters observed with disk A were generally 2 mm lower than those observed with disk B, independent of the MHA used. Despite this difference, all inhibition zones for QC strain S. aureus ATCC 25923 were within CLSI-acceptable ranges, and error rates were within acceptable ranges for both disk reagents when the results were analyzed for each disk reagent separately.

This study showed that CC5 isolates were dominant among the ceftaroline-nonsusceptible group (MIC, ≥2 μg/ml), and these isolates originated mostly from countries in the Asia-Pacific region. A high prevalence of the CC5 lineage among MRSA isolates causing infections in Asian countries, as well as among isolates displaying ceftaroline MIC values of ≥2 μg/ml, has been reported by other investigators (6, 22, 23), and these results suggest that isolates belonging to this lineage may possess competitive survival advantages over other MRSA lineages under selective pressure in the hospital environment (24).

Isolates having a wild-type PBP2a exhibited ceftaroline MIC results (1 to 4 μg/ml) that overlapped with those carrying single (1 to 4 μg/ml) or multiple (2 to 16 μg/ml) mutations; although overlapping MIC results were observed, the ceftaroline modal MIC values for wild-type and PBP2a mutant strains were very distinct. While wild-type isolates had ceftaroline modal MIC results of 1 μg/ml, isolates with PBP2a alterations demonstrated modal MIC values of either 2 or 4 μg/ml, depending on the mutation position (allosteric versus transpeptidase sites). Nevertheless, despite distinct modal MIC results, the overlapping MIC values between isolates with wild-type molecular results and those with nucleotide/amino acid alterations (non-wild type) challenge the separation between these 2 groups by the reference broth microdilution and disk diffusion methods. It is also important to note that 4 molecularly characterized isolates with wild-type sequences for PBP2a and the upstream region of *pbp4* showed reproducible ceftaroline MIC values of 2 to 4 μg/ml, suggesting that other mechanisms for decreased susceptibility may be present.

The analysis of the correlation (or lack thereof) between disk inhibition zones and PBP2a alterations clearly showed that the disk diffusion test was also not able to satisfactorily separate the group of isolates with no molecular alterations from those with alterations, or between those having alteration(s) at different regions. Overall, among isolates having alteration(s) at both allosteric and transpeptidase sites and isolates having alteration(s) at the transpeptidase domain, 18.8% and 37.5% were categorized as susceptible by the disk diffusion method, respectively. In contrast, 0.0% and 14.3% (1 isolate with a K565N mutation) were categorized, respectively, as susceptible by the broth microdilution method (Tables 4 and 5).

The molecular results and the interpretation of the results presented here are important cofounding variables with regard to their alterations or combinations, and the location (nPBD versus PBD) of any alteration(s) should be considered wild type (6). Several examples presented here illustrate scenarios where wild-type MRSA isolates had ceftaroline MIC results of 2 to 4 μg/ml in the SDD category, while an isolate carrying an alteration at the PBD of PBP2a (K565N) had a reproducible ceftaroline MIC value of 1 μg/ml. Several PBP2a alterations detected here were reported previously and were associated with isolates having ceftaroline MIC results of ≥2 μg/ml (6, 25–27). However, the relevance of PBP2a mutations, such as K565N (ceftaroline MIC of 1 μg/ml and zone diameters ranging from 22 to 28 mm), at the transpeptidase site remains unknown. The K565 residue is located far from the transpeptidase pocket, and it is unlikely that a variation at this position impacts ceftaroline binding. The isolate showing a K565N mutation also had a nucleotide substitution upstream of the *pbp4* gene, which could result in higher expression of this gene and, consequently, the ceftaroline MIC value (1 μg/ml) at the end of the wild-type distribution (20, 25). Therefore, the broth microdilution method correctly characterized this isolate as susceptible, and 3 out 4 disk diffusion results were also correctly assigned as susceptible.

In summary, the results from the disk diffusion method showed a good correlation with those from the reference broth microdilution method, with low discrepancy rates, when testing ceftaroline against a challenge collection of MRSA isolates. Furthermore, the results of this investigation corroborate those of previous publications by showing that the ceftaroline MIC distribution of wild-type MRSA isolates, i.e., isolates with no mutations in PBP2a, goes up to 4 μg/ml, and reference broth microdilution or disk diffusion methods do not properly separate wild-type from non-wild-type populations using current breakpoints (6, 25, 26).
